# Supplementary material for: Role and mechanism of NCAPD3 in promoting malignant behaviors in gastric cancer
Source: Front Pharmacol. 2024 Apr 22;15:1341039. doi: 10.3389/fphar.2024.1341039 (PMC11070777; doi:10.3389/fphar.2024.1341039)
Supplement: Supplementary file 11 [file DataSheet2.ZIP › GSEA/GO_CC/my_analysis.Gsea.1599461408869/GO_SOMATODENDRITIC_COMPARTMENT.html]

Details for gene set GO\_SOMATODENDRITIC\_COMPARTMENT[GSEA]

|  || Dataset | filtered\_dataset.sample\_info.cls#WT\_versus\_NCAPD3\_MUT |
| Phenotype | sample\_info.cls#WT\_versus\_NCAPD3\_MUT |
| Upregulated in class | WT |
| GeneSet | GO\_SOMATODENDRITIC\_COMPARTMENT |
| Enrichment Score (ES) | 0.22808923 |
| Normalized Enrichment Score (NES) | 1.3471688 |
| Nominal p-value | 0.11556982 |
| FDR q-value | 0.5399726 |
| FWER p-Value | 1.0 |
Table: GSEA Results Summary

  

Fig 1: Enrichment plot: GO\_SOMATODENDRITIC\_COMPARTMENT      
 Profile of the Running ES Score & Positions of GeneSet Members on the Rank Ordered List

  

| SYMBOL | TITLE | RANK IN GENE LIST | RANK METRIC SCORE | RUNNING ES | CORE ENRICHMENT || 1 | 659 | BMPR2 | 16 | 1.047 | 0.0220 | Yes |
| 2 | 6421 | SFPQ | 87 | 0.818 | -0.0033 | Yes |
| 3 | 7976 | FZD3 | 88 | 0.815 | 0.0231 | Yes |
| 4 | 56288 | PARD3 | 99 | 0.790 | 0.0412 | Yes |
| 5 | 5967 | REG1A | 100 | 0.788 | 0.0666 | Yes |
| 6 | 23380 | SRGAP2 | 142 | 0.757 | 0.0608 | Yes |
| 7 | 3770 | KCNJ14 | 157 | 0.721 | 0.0737 | Yes |
| 8 | 613 | BCR | 180 | 0.695 | 0.0800 | Yes |
| 9 | 9897 | KIAA0196 | 197 | 0.682 | 0.0902 | Yes |
| 10 | 9456 | HOMER1 | 198 | 0.680 | 0.1121 | Yes |
| 11 | 26278 | SACS | 205 | 0.669 | 0.1293 | Yes |
| 12 | 8831 | SYNGAP1 | 236 | 0.647 | 0.1280 | Yes |
| 13 | 57521 | RPTOR | 239 | 0.645 | 0.1474 | Yes |
| 14 | 23001 | WDFY3 | 240 | 0.642 | 0.1681 | Yes |
| 15 | 5701 | PSMC2 | 244 | 0.636 | 0.1864 | Yes |
| 16 | 55238 | SLC38A7 | 250 | 0.630 | 0.2031 | Yes |
| 17 | 1161 | ERCC8 | 255 | 0.625 | 0.2203 | Yes |
| 18 | 5728 | PTEN | 285 | 0.606 | 0.2185 | Yes |
| 19 | 538 | ATP7A | 305 | 0.588 | 0.2234 | Yes |
| 20 | 6715 | SRD5A1 | 325 | 0.578 | 0.2281 | Yes |
| 21 | 9743 | ARHGAP32 | 413 | 0.528 | 0.1809 | No |
| 22 | 23242 | COBL | 426 | 0.516 | 0.1887 | No |
| 23 | 6507 | SLC1A3 | 464 | 0.496 | 0.1774 | No |
| 24 | 3836 | KPNA1 | 548 | 0.448 | 0.1307 | No |
| 25 | 10445 | MCRS1 | 617 | 0.414 | 0.0938 | No |
| 26 | 2021 | ENDOG | 648 | 0.400 | 0.0846 | No |
| 27 | 57679 | ALS2 | 656 | 0.399 | 0.0923 | No |
| 28 | 2932 | GSK3B | 714 | 0.367 | 0.0621 | No |
| 29 | 3323 | HSP90AA4P | 793 | 0.329 | 0.0152 | No |
| 30 | 3312 | HSPA8 | 848 | 0.244 | -0.0168 | No |
| 31 | 55450 | CAMK2N1 | 861 | -0.269 | -0.0170 | No |
| 32 | 6515 | SLC2A3 | 873 | -0.283 | -0.0160 | No |
| 33 | 9181 | ARHGEF2 | 902 | -0.326 | -0.0261 | No |
| 34 | 23710 | GABARAPL1 | 913 | -0.341 | -0.0225 | No |
| 35 | 3949 | LDLR | 929 | -0.353 | -0.0222 | No |
| 36 | 2317 | FLNB | 936 | -0.360 | -0.0150 | No |
| 37 | 81544 | GDPD5 | 961 | -0.381 | -0.0204 | No |
| 38 | 5621 | PRNP | 970 | -0.387 | -0.0138 | No |
| 39 | 9572 | NR1D1 | 977 | -0.392 | -0.0056 | No |
| 40 | 23046 | KIF21B | 1034 | -0.433 | -0.0329 | No |
| 41 | 80315 | CPEB4 | 1053 | -0.448 | -0.0318 | No |
| 42 | 1191 | CLU | 1056 | -0.450 | -0.0187 | No |
| 43 | 5818 | PVRL1 | 1073 | -0.458 | -0.0157 | No |
| 44 | 283 | ANG | 1074 | -0.458 | -0.0009 | No |
| 45 | 9939 | RBM8A | 1093 | -0.474 | 0.0011 | No |
| 46 | 1263 | PLK3 | 1134 | -0.502 | -0.0122 | No |
| 47 | 7074 | TIAM1 | 1143 | -0.508 | -0.0017 | No |
| 48 | 3706 | ITPKA | 1150 | -0.514 | 0.0105 | No |
| 49 | 54407 | SLC38A2 | 1182 | -0.553 | 0.0055 | No |
| 50 | 4651 | MYO10 | 1213 | -0.588 | 0.0023 | No |
| 51 | 2043 | EPHA4 | 1228 | -0.602 | 0.0114 | No |
| 52 | 54800 | KLHL24 | 1287 | -0.677 | -0.0095 | No |
| 53 | 54464 | XRN1 | 1301 | -0.703 | 0.0036 | No |
| 54 | 885 | CCK | 1302 | -0.703 | 0.0263 | No |
| 55 | 6509 | SLC1A4 | 1330 | -0.752 | 0.0307 | No |
| 56 | 4734 | NEDD4 | 1369 | -0.856 | 0.0303 | No |
Table: GSEA details [plain text format]

  

Fig 2: GO\_SOMATODENDRITIC\_COMPARTMENT      
 Blue-Pink O' Gram in the Space of the Analyzed GeneSet

  

Fig 3: GO\_SOMATODENDRITIC\_COMPARTMENT: Random ES distribution      
 Gene set null distribution of ES for **GO\_SOMATODENDRITIC\_COMPARTMENT**
